# Subjective social status and common mental disorders in the Swedish working population: gender and age differences in longitudinal associations

**DOI:** 10.1177/14034948251380490

**Published:** 2026-02-17

**Authors:** Ylva Lindberg, Erik Grönqvist, Åsa Andersén, Kristiina. Rajaleid, Göran Kecklund, Anna Nyberg

**Affiliations:** 1 Department of Public Health and Caring Sciences, Uppsala University, Uppsala, Sweden; 2 Department of Medical Sciences, Health Economics, Uppsala University, Uppsala, Sweden; 3 Centre for Health Economic Research (HEFUU), Uppsala University, Uppsala, Sweden; 4 Department of Psychology, Stockholm University, Stockholm, Sweden; 5 Department of Public Health Science, Stockholm University, Stockholm, Sweden

**Keywords:** Women, mental health, depression, sleep disturbances, fixed effects

## Abstract

**Aims:**

To investigate predictors and common mental disorder outcomes of subjective social status in different gender and age groups of Swedish employees.

**Methods:**

Data from eight waves of the Swedish Longitudinal Occupational Survey of Health (SLOSH), collected between 2006 and 2020, were used (*n*=12,925). Fixed effects models were used to investigate whether changes in demographic and socioeconomic factors predicted changes in subjective social status and whether changes in subjective social status, in turn, affected symptoms of depression and sleep disturbances. The analyses were stratified by gender (female/male) and age (21–35/36–50 years).

**Results:**

Across groups, the most consistent predictors of changes in subjective social status were being promoted during the past two years and a change in occupational status. An increase in subjective social status was associated with a decrease in symptoms of depression among women in both age groups and among men in the older age group. The association was stronger in younger women than in older women (*p*=0.042). For both women and men in the older age group, an increase in subjective social status was associated with a decrease in symptoms of sleep disturbances.

**Conclusions:**

Occupational level was the most important predictor of subjective social status across groups, and subjective social status predicted common mental disorder, even after adjusting for socioeconomic status indicators. Changes in subjective social status affected young working women’s depressive symptoms in particular, while older workers showed an association between subjective social status and symptoms of sleep disturbances.

## Background

Common mental disorders (CMDs) such as depression and stress-related disorders have increased in recent years in Sweden, particularly among young women [1]. The reason for this increase, or for the gender difference, is not clear. CMDs are a major cause of sickness absence among young women [2], and limited research is available on risk and protective factors. One acknowledged predictor of CMDs is an individual’s position in a status hierarchy, measured in terms of socioeconomic status (SES) or a subjective perception of one’s status position relative to others; that is, subjective social status (SSS) [3]. SSS is influenced by objective measures of SES, such as education, occupation and income, but research has consistently found that SSS captures aspects over and beyond the SES indicators [4].

The association between SSS and mental health has been well documented. In a recent review, lower SSS was found to be associated with poorer mental health and lower wellbeing [3]. SSS has been associated with both depressive symptoms [5–7] and impaired sleep quality [5, 8]. Depressive symptoms and sleep disturbances are prevalent health complaints among Swedish employees [9]. In studies controlling for SES, the associations between SSS and health remained, indicating an independent effect of SSS [4]. A key methodological challenge is controlling for confounding when investigating the health effects of SSS. Using panel data and fitting fixed effects regression models has been suggested, thus controlling for all time-invariant characteristics [10].

Previous studies investigating the moderating role of gender and age in the associations between SSS and mental health among various groups show an inconclusive picture [6–8, 11, 12]. There also appears to be a lack of studies focusing on the group of young working women. This group might differ from others, for instance, in the way they are exposed to the possibility of social comparison through social media, in which young women especially report negative experiences [13]. Women may, furthermore, compare themselves in more areas than men [14], while at the same time facing greater difficulty in establishing themselves in higher positions in the labour market [15] – partly due to poorer psychosocial work environments with, for example, high demands and low decision authority, where many women are employed [16]. The impact of low SSS may, therefore, be particularly high on symptoms of CMDs among women in the years of establishment in the labour market.

The importance of different indicators for ratings of SSS may differ by demographics such as ethnicity [17] and gender [17, 18]. Workers in younger age groups (up to approximately 35 years of age) are often trying to establish themselves in the labour market. This is also a time when many are forming families. Younger workers may thus be experiencing less stability than older workers. Young working women may also differ from their male counterparts due to young men and women often entering different labour market sectors when entering working life [19] and women facing the constraint of fewer reproductive years. Predictors of SSS are therefore likely to differ for young working women compared with other groups in the labour market. However, limited research has specifically examined this group, and knowledge about the predictors and consequences of SSS for young working women remains, to the best of our knowledge, largely unexplored.

## Aim

The aim was to investigate predictors and CMD outcomes of SSS in different gender and age groups of Swedish employees.

### Research questions

1. Are changes in demographic and SES factors over time associated with individual and group changes in SSS in women and men in young adulthood (21–35 years) and adulthood (36–50 years)?
2. Is SSS associated with symptoms of: (a) depression; and (b) sleep disturbances in men and women in young adulthood and adulthood? Do these associations differ by gender and age group?

## Methods

### Data

This study used data from the Swedish Longitudinal Occupational Survey of Health (SLOSH), a cohort derived from a nationally representative sample of the Swedish working population. The SLOSH data comprise information on working conditions, social situation, health and wellbeing [20, 21]. Survey data collection and linking to register data were performed by Statistics Sweden on behalf of Stockholm University. Survey data were collected biennially in 2006–2022, and then yearly. In this study, data from eight waves (2006–2020) were used. Pseudonymised, project-specific data were released to Uppsala University. The overall response rate varied between 65% and 48%. The present study included 12,925 SLOSH participants aged 21–50 years, who had answered 2.32 questionnaires on average, with complete data.

### Measures

#### Subjective social status

SSS was measured using a survey question asking respondents to place themselves on a ten-rung ladder illustrating social status: At the top are the people who are the best off (most money, most education, best jobs) and at the bottom those who are the worst off (least money, least education, worst or no job). SSS was used as a continuous variable with values of 1–10. It was used as an outcome variable (research question 1) and an exposure variable (research question 2).

#### Symptoms of CMD

Symptoms of depression were measured using the symptom checklist–core depression (SCL–CD_6_) scale [22], utilising six items capturing different types of depressive sentiments over the past week (lethargy; depressed; self-blame; worrying; no interest; everything being strenuous). Answers were reported on a Likert scale from 1 (not at all) to 5 (extremely), summed to form an index ranging from 6 to 30, and transformed into a standardised continuous variable (mean = 0, standard deviation (SD) = 1) within each survey wave.

Symptoms of sleep disturbances were measured with six items from the Karolinska sleep questionnaire (KSQ) [23], capturing different types of sleeping problems during the past 3 months (difficulties falling asleep; difficulties waking up; repeated awakenings; not rested at awakening; premature awakening; unrestful sleep), reported on a Likert scale from 1 (never) to 6 (always). The answers were summed to form an index ranging from 6 to 36, and transformed into a standardised continuous variable (mean = 0, SD = 1) within each survey wave.

Cronbach’s alpha for depressive symptoms was 0.9085, and for symptoms of sleep disturbances it was 0.8270.

#### Demographic and SES factors

Sociodemographic factors used were survey-based indicators of marital status, children living at home and, from registry data, age in 5-year categories.

Income was measured from registry data as annual income in Swedish crowns, divided into quintiles within each wave and analysed as a continuous variable.

Occupation by first-digit occupational code in the Swedish version of the ISCO-08 classification (International Standard Classification of Occupations) was based on a survey question. The ISCO-08 is hierarchical in terms of skill level and specialization [24]; occupation is thereby treated as a continuous variable, with higher values denoting lower skill levels.

Educational attainment recorded respondents’ highest level of education, based on registry data, and was analysed as a continuous variable.

Being promoted or demoted during the past 2 years was measured with a survey question asking the respondent whether they had got a different position during the past 2 years, with possible answers ‘yes, higher’, ‘no, unchanged’, or ‘yes, lower’. ‘Yes, higher’ was coded as promotion and ‘yes, lower’ was coded as demotion, and then used as two separate categorical variables.

#### Age and gender

The sample was divided into two age groups, 21–35 years and 36–50 years, representing younger adults who are establishing themselves in the labour market and adults already established – a categorisation used previously [25].

Gender was a dichotomous, registry-based variable (female/male).

### Analytical strategy

In a descriptive analysis, correlates to SSS were explored by regressing the SSS score jointly on demographic and SES factors. To investigate how changes in demographic and SES factors predict a changed SSS, individual fixed effects were included in the multiple regression analysis. The influence of SSS on symptoms of depression and sleep disturbances was analysed using fixed effects models [26]. Outcomes were compared between those with and those without a changed level of SSS over subsequent survey waves. We thus identify the parameter of interest – that is, how SSS impacts symptoms of depression or sleep disturbances. By comparing individuals’ outcomes over time, all time-invariant individual-specific (observable and unobservable) confounders were differenced out, and by comparing with other individuals not experiencing a (similar) change in SSS, any general trend in outcomes was accounted for. Our empirical strategy relies on a model with individual and time-fixed effects, time-varying covariates, and heteroskedasticity-robust standard errors to address potential endogeneity, control for observable and unobservable confounders, and ensure valid inference.

The following linear regression model (OLS) was estimated for individual *i* measured in calendar year *w*:

$$y_{iw}=\alpha_{i}+\alpha_{w}+\beta {\mathrm{SSS}}_{iw}+\gamma x_{iw}+\varepsilon_{iw},$$

where $y_{iw}$ is the outcome; that is, symptoms of depression or sleep disturbances. The intercept $\alpha_{i}$ denotes the individual fixed effect, capturing all individual-level confounders that are constant over time (e.g. personality or SES background), $\alpha_{w}$ is biennial calendar time (i.e. survey wave) fixed effects, that non-parametrically capture all time-varying factors that are shared across individuals (e.g. general trends in society), and γ captures the influence of observed individual-level, time-varying confounders $x_{iw}$ . The exposure, that is, SSS, is denoted by SSS_*iw*_. The parameter of interest is β . Under the identifying assumptions that individuals who experience a decreased (increased) SSS would have had the same change in outcomes, in the absence of the decreased (increased) SSS, as individuals not experiencing a changed SSS (parallel trends), β describes how the change in SSS, on average, influences the level of symptoms of depression and sleep disturbances. Moreover, changes in SSS must not be generated by depression or sleep disturbances (reverse causality).

Measurement error is an inherent concern in self-reported survey data. Classic errors in the independent variable may attenuate estimates towards zero, whereas errors in the dependent variable increase variance but do not bias the coefficient; fixed effects for individuals and survey waves help mitigate bias from time or person-specific misreporting.

To assess the validity of the identifying assumptions, an unadjusted model (model 1), only including individual and survey-wave fixed effects, was first estimated. In model 2, individual time-varying characteristics (marital status, 5-year age classes, and having children living at home) were added as controls, and in the final, full model (model 3), SES indicators (income quintile, educational attainment, and occupation) were also included. Robust standard errors were clustered on individuals. To test for differences in the impact of SSS between groups, a fully interacted regression was estimated, in which the difference in estimates for ‘older’ and ‘men’ was tested against the base category of ‘younger’ and ‘women’.

Results are presented with 95% confidence intervals (CIs). STATA version 17 was used for all analyses. The Strengthening the Reporting of Observational Studies in Epidemiology (STROBE) cohort guidelines were used to report the findings [27].

### Ethical considerations

The project received ethical approval from the Swedish Ethical Review Authority (approval no. 2022-00417-01).

## Results

### Sample characteristics

Women in both age groups had a higher mean score for symptoms of depression (0.37 and 0.23, respectively) than men (0.08 and 0.01, respectively). For symptoms of sleep disturbances, a similar pattern was observed, with women in both age groups having higher mean scores (0.17 and 0.16, respectively) than men (0.01 and −0.03, respectively). Mean scores for SSS were similar for all groups, ranging between 6.20 and 6.41 (Table I).

**Table I.** Sample characteristics.

	Age 21–35 years		Age 36–50 years	
Variable	Women	Men	Women	Men
Depressive symptoms (Mean (SD))	0.37 (1.10)	0.08 (0.96)	0.23 (1.08)	0.01 (0.97)
Sleep disturbances (Mean (SD))	0.17 (0.99)	0.01 (0.91)	0.16 (1.01)	−0.03 (0.94)
SSS (Mean (SD))	6.20 (1.46)	6.36 (1.47)	6.29 (1.56)	6.41 (1.60)
Married/cohabiting (%)	77.2	74.2	81.2	81.7
Job position				
Promoted (%)	30.6	33.6	22.0	24.3
Demoted (%)	2.7	2.4	3.3	3.1
Occupation (%)				
1 Managers	2.9	5.5	8.4	12.4
2 Occupations requiring advanced level of higher education	37.5	26.8	38.6	26.5
3 Occupations requiring higher education qualifications or equivalent	22.5	22.2	19.8	23.4
4 Administration and customer service clerks	9.4	4.2	10.4	3.9
5 Service, care and shop sales workers	19.7	9.6	17.7	5.8
6 Agricultural, horticultural, forestry and fishery workers	1.1	2.1	0.6	1.5
7 Building and manufacturing workers	1.2	15.7	0.8	13.8
8 Mechanical manufacturing and transport workers, etc.	2.6	11.9	1.6	11.2
9 Elementary occupations	3.2	2.0	2.1	1.5
Age category, years (%)				
21–25	6.2	5.0		
26–30	29.9	25.4		
31–35	63.9	69.6		
36–40			23.7	24.1
41–45			34.0	34.0
45–50			42.3	42.0
Children at home (%)	48.6	45.7	78.7	76.4
Born in Sweden (%)	96.3	96.5	93.5	94.4
Income quintile (%)				
1 (lowest)	41.7	16.1	16.1	5.5
2	26.9	18.2	25.7	10.9
3	15.8	24.6	23.3	19.8
4	11.0	24.7	18.7	27.6
5 (highest)	4.7	16.4	16.2	36.2
Educational att. (%)				
Elementary	1.5	3.1	2.5	4.7
Secondary	31.4	46.3	35.0	45.4
Short higher education	5.8	5.7	6.1	10.2
University	61.3	44.9	56.4	39.7
Observations (*n*)	3212	2318	14,161	10,392
Individuals (*n*)	1904	1424	6325	4962

SD: standard deviation; SSS: subjective social status.

### Descriptive analysis of demographic and SES factors and SSS

For all groups, being promoted in the past 2 years, being married or cohabiting, higher income, and higher education were positively associated with SSS, while occupation (in which higher values denote lower skill level of the occupation) was negatively associated with SSS. Being demoted was only associated with SSS for men aged 36–50 years (−0.38, 95% CI −0.57 to −0.18), and having children living at home was only associated with SSS for men aged 21–35 years (−0.17, 95% CI −0.31 to −0.02). Age was associated with SSS for both women and men aged 21–35 years, but in different directions (0.14, 95% CI 0.04 to 0.24 and −0.12, 95% CI −0.24 to −0.00, respectively) (Table II).

**Table II.** Association between demographic and SES factors and SSS.

	Age 21–35 years		Age 36–50 years	
Variable	Women Coeff. (CI)	Men Coeff. (CI)	Women Coeff. (CI)	Men Coeff. (CI)
Promoted	0.35 (0.25–0.45)	0.32 (0.20–0.43)	0.23 (0.18–0.29)	0.20 (0.14–0.26)
Demoted	−0.14 (−0.44–0.17)	−0.07 (−0.54–0.41)	−0.06 (−0.19–0.08)	−0.38 (−0.57– −0.18)
Married/cohabiting	0.45 (0.32–0.59)	0.27 (0.11–0.43)	0.53 (0.45–0.61)	0.35 (0.24–0.45)
Born in Sweden	0.13 (−0.17–0.43)	0.06 (−0.32–0.44)	0.09 (−0.03–0.22)	0.03 (−0.13–0.18)
Children living at home	−0.06 (−0.18–0.06)	−0.17 (−0.31– −0.02)	−0.06 (−0.13–0.02)	0.02 (−0.07–0.11)
Age category	0.14 (0.04–0.24)	−0.12 (−0.24–−0.00)	−0.02 (−0.04–0.02)	0.03 (−0.01–0.06)
Income quintile	0.22 (0.18–0.27)	0.28 (0.23–0.33)	0.22 (0.20– 0.24)	0.28 (0.25–0.31)
Educational attainment	0.25 (0.18–0.32)	0.20 (0.11–0.28)	0.12 (0.09–0.16)	0.08 (0.04–0.12)
Occupation	−0.11 (−0.15– −0.07)	−0.08 (−0.12– −0.05)	−0.13 (−0.16– −0.11)	−0.13 (−0.15– −0.12)
Observations	3099	2252	13,261	9759
Individuals	1863	1406	6100	4792
*R* ^2^	0.24	0.21	0.28	0.31

CI: confidence interval; SES: socioeconomic status; SSS: subjective social status.

The table reports regressing SSS and different characteristics. All models include survey wave fixed effects.

### Associations between changes in demographic and SES factors, and changes in SSS

Being promoted in the past 2 years was associated with an increase in SSS in the older age groups. Getting married or starting cohabiting was associated with an increase in SSS for women aged 36–50 years, and increased income was associated with an increase in SSS for men aged 36–50 years. A change to a lower status occupation was associated with a decrease in SSS for women aged 21–35 years and for men aged 36–50 years (Table III).

**Table III.** Within individual association between change in demographic and SES indicators and change in SSS.

	Age 21–35 years		Age 36–50 years	
Variable	Women Coeff. (CI)	Men Coeff. (CI)	Women Coeff. (CI)	Men Coeff. (CI)
Promoted	0.19 (−0.02–0.39)	0.24 (−0.04–0.51)	0.15 (0.08–0.23)	0.12 (0.03–0.20)
Demoted	−0.59 (−1.21–0.03)	0.33 (−0.52–1.18)	−0.06 (−0.24–0.13)	−0.07 (−0.32–0.19)
Married/cohabiting	0.19 (−0.11–0.50)	0.12 (−0.31–0.55)	0.21 (0.05–0.37)	0.08 (−0.12–0.27)
Children living at home	−0.09 (−0.40–0.22)	−0.09 (−0.53–0.35)	−0.03 (−0.17–0.11)	0.02 (−0.17–0.21)
Age category	−0.06 (−0.32–0.20)	−0.05 (−0.38–0.27)	0.01 (−0.08–0.09)	−0.00 (−0.11–0.10)
Income quintile	0.02 (−0.08–0.13)	−0.03 (−0.16–0.10)	0.03 (−0.02–0.07)	0.08 (0.01–0.14)
Educational attainment	0.42 (−0.04–0.87)	0.16 (−0.34–0.65)	0.20 (−0.12–0.51)	0.27 (−0.12–0.65)
Occupation	−0.13 (−0.23– −0.03)	−0.05 (−0.16–0.06)	−0.04 (−0.09–0.01)	−0.07 (−0.11– −0.03)
Observations	3100	2252	13,264	9762
Individuals	1863	1406	6100	4793
*R* ^2^	0.84	0.85	0.81	0.83

CI: confidence interval; SES: socioeconomic status; SSS: subjective social status.

The table reports regressing SSS and different characteristics. All models include individual and survey wave fixed effects.

### Association between changes in SSS and symptoms of depression

In the fully adjusted model (model 3), there was an association between a change in SSS and a change in symptoms of depression for women in both age groups and for men aged 36–50 years, but not for men aged 21–35 years. The results indicate that, for women aged 21–35 years, an increase of one rung on the ladder scale for SSS was associated with a decrease of 0.09 (95% CI −0.17 to −0.01) SDs in depression score, and for women and men aged 36–50 years, a decrease of 0.04 (95% CI −0.07 to −0.01) and 0.04 (95% CI −0.07 to −0.02), respectively (Table IV).

**Table IV.** Fixed effects models for the association between SSS and symptoms of depression.

	Age 21–35 years		Age 36–50 years	
	Women *N*_observations_ = 3185 *N*_individuals_ = 1893	Men *N*_observations_ = 2304 *N*_individuals_ = 1481	Women *N*_observations_ = 14,036 *N*_individuals_ = 6291	Men *N*_observations_ = 10,311 *N*_individuals_ = 4944
Model 1	−0.09 (−0.17– −0.01)	−0.04 (−0.13–0.05)	−0.04 (−0.07– −0.01)	−0.04 (−0.07– −0.01)
*R* ^2^	0.80	0.82	0.74	0.78
Model 2	−0.09 (−0.17– −0.01)	−0.04 (−0.13–0.05)	−0.04 (−0.07– −0.01)	−0.04 (−0.07– −0.01)
*R* ^2^	0.80	0.82	0.74	0.78
Model 3	−0.09 (−0.17–0.01)	−0.03 (−0.12–0.06)	−0.04 (−0.07– −0.01)	−0.04 (−0.07– −0.02)
*R* ^2^	0.80	0.82	0.74	0.78

SSS: subjective social status.

The table reports regressing a normalised depression index on SSS. All models include individual and survey wave fixed effects. Model 1 is the unadjusted model. Model 2 controls for indicators for marital status, 5-year age classes, and having children living at home. Model 3 additionally controls for indicators of income quintile, educational attainment, and one digit occupational code (ISCO).

The only difference found between groups was between women aged 21–35 and 36–50 years (*p*=0.042 for model 3), indicating a larger effect between a change in SSS and symptoms of depression for the younger age group in women (see Supplemental material).

### Association between changes in SSS and symptoms of sleep disturbances

For the older age groups, there was an association between SSS and symptoms of sleep disturbances in the fully adjusted model. For the younger age groups, there was no such association. The results indicate that, for women and men aged 36–50 years, an increase of one rung on the ladder scale for SSS was associated with a decrease of 0.05 (95% CI −0.07 to −0.03) and 0.06 (95% CI −0.09 to −0.03) SDs in symptoms of sleep disturbances score, respectively (Table V).

**Table V.** Fixed effects models of the association between SSS and symptoms of sleep disturbances.

	Age 21–35 years		Age 36–50 years	
	Women *N*_observations_ = 3065 *N*_individuals_ = 1861	Men *N*_observations_ = 2244 *N*_individuals_ = 1404	Women *N*_observations_ = 12,803 *N*_individuals_ = 5956	Men *N*_observations_ = 9458 *N*_individuals_ = 4695
Model 1	−0.08 (−0.14– −0.01)	−0.06 (−0.14–0.03)	−0.05 (−0.07– −0.03)	−0.06 (−0.09– −0.03)
*R* ^2^	0.84	0.86	0.81	0.83
Model 2	−0.07 (−0.14– −0.00)	−0.06 (−0.14–0.03)	−0.05 (−0.07– −0.03)	−0.06 (−0.09– −0.03)
*R* ^2^	0.84	0.86	0.81	0.83
Model 3	−0.06 (−0.13–0.00)	−0.06 (−0.14–0.03)	−0.05 (−0.07– −0.03)	−0.06 (−0.09– −0.03)
*R* ^2^	0.84	0.86	0.81	0.83

SSS: subjective social status.

The table reports regressing a normalised sleep disturbances index on SSS. All models include individual and survey wave fixed effects. Model 1 is the unadjusted model. Model 2 controls for indicators for marital status, 5-year age classes, and having children living at home. Model 3 additionally controls for indicators of income quintile, educational attainment, and one digit occupational code (ISCO).

There were no statistically significant differences between groups regarding associations between changes in SSS and symptoms of sleep disturbances (see Supplemental material).

## Discussion

The aim was to investigate predictors and CMD outcomes of SSS in different gender and age groups of the Swedish working population. Results showed that being promoted during the past 2 years and a change in occupational status were the most consistent predictors of SSS. An increase in SSS was associated with a decrease in symptoms of depression in women in both age groups and in men in the older age group. The association was stronger in younger women than in older women. For the older age groups, there was an association between an increase in SSS and a decrease in symptoms of sleep disturbances, with no difference between groups.

### Predictors of changes in SSS

For women aged 21–35 years, the only predictor of SSS was a change in occupational status. An earlier study concluded that, although not exclusively, traditional SES measures such as occupation are important determinants of SSS [28]. This association was also present for men aged 36–50 years, suggesting there might be different mechanisms involved, the first group being in the process of establishing themselves in both working and private life, and the groups having different genders. Women face more difficulty in establishing themselves in higher positions in the labour market [15] and therefore might be more affected by a change in occupational status. Based on the data, it is not possible to determine what circumstances preceded the change in occupational status. However, this association was not observed for women aged 36–50 years, indicating possible mechanisms inherent in the earlier stages of working life, such as more insecure job positions. Women in the older age group might also be more established in other areas of life and thus be less affected by work-life factors.

### Associations between SSS and symptoms of depression

Both groups of women had an association between an increase in SSS and a decrease in symptoms of depression. However, the association was stronger in the younger group, suggesting that age plays a role in the relationship between SSS and symptoms of depression among women. This contrasts with earlier studies on adolescents [11] but aligns with research showing associations between age, SSS, and depression among the elderly [6]. The current findings, based on different age groups than those examined in previous studies, contribute to a broader understanding of how age influences the relationship between SSS and mental health. The present study cannot determine the underlying reason for the stronger association in the younger group. Potential explanations include younger women still being in the process of establishing themselves across various domains of life, and thus could be more sensitive to social comparisons and changes in their perceived social status.

### Associations between SSS and symptoms of sleep disturbances

There was an association between SSS and symptoms of sleep disturbances in the fully adjusted model only for the older age group. The difference in comparison with the younger age group was, however, not statistically significant. Lower SSS has been found to be a predictor of short sleep duration in an earlier longitudinal study [29]; however, the dimension of age was not addressed. In the present study, this dimension is added, contributing to the knowledge on the relationship between SSS and sleep disturbances.

### Strengths and limitations

Strengths include the focus on an under-researched age group in the occupational literature, the use of eight waves of a large set of panel data based on a representative sample of the Swedish working population, as well as several measures from registers with a high proportion of complete data. The use of fixed effects modelling accounts for unobservable time-invariant differences across individuals, and time-varying confounding is addressed with control variables. Results are (qualitatively) stable to including controls, indicating that the assumption of parallel trends holds [30].

However, some limitations can be noted. Selective attrition in the SLOSH cohort limits generalisability to the Swedish working population. Furthermore, both SSS and CMD indicators were self-reported, with risks of inflated associations due to common method bias. Data thus suffer from heterogeneity with respect to the underlying SSS and CMD, but to the extent that responses have a high internal consistency, the fixed effects design accounts for such heterogeneity. Another limitation is that the fixed effects design, comparing individuals over time, builds on changes in SSS and outcomes, thus using less variation than cross-sectional studies.

## Conclusions

The most important predictor of SSS across groups appears to be occupational level. A change in SSS seems to affect young working women’s depressive symptoms in particular, while older workers showed an association between SSS and symptoms of sleep disturbances. A change in SSS predicted CMD, even after adjustment for SES indicators.

The results suggest that efforts to strengthen young women’s occupational positions are important. Future studies could investigate further the reasons behind and mechanisms involved in changing occupational status and its relationship to SSS. To generate in-depth knowledge about young working women’s perceptions, qualitative methods could be employed.
